# Update on prevalence of diagnosed systemic lupus erythematosus (SLE) by major health insurance types in the US in 2016

**DOI:** 10.1186/s13104-021-05877-1

**Published:** 2022-01-09

**Authors:** Yiting Wang, Laura L. Hester, Jennifer Lofland, Shawn Rose, Chetan S. Karyekar, David M. Kern, Margaret Blacketer, Kourtney Davis, Kimberly Shields-Tuttle

**Affiliations:** 1 Janssen Research & Development, LLC, 1125 Trenton-Harbourton Road, Titusville, NJ 08560 USA; 2 Janssen Scientific Affairs, LLC, Horsham, PA USA; 3 Janssen Research & Development, LLC, Spring House, PA USA

**Keywords:** Systemic lupus erythematosus, Health insurance, Prevalence, Epidemiology

## Abstract

**Objective:**

To provide current estimates of the number of patients with prevalent systemic lupus erythematosus (SLE) by major health insurance types in the US and to describe patient characteristics. Four large US health insurance claims databases were analyzed to represent different types of insurance coverage, including private insurance, Medicaid, and Medicare Supplemental.

**Results:**

Overall unadjusted SLE prevalence per 100,000 persons in the US ranged from 150.1 (private insurance) to 252.9 (Medicare Supplemental insurance). Extrapolating to the US civilian population in 2016, we estimated roughly 345,000 to 404,000 prevalent SLE patients with private/Medicare insurance and 99,000 prevalent SLE patients with Medicaid insurance. Comorbidities, including renal failure/dialysis were commonly observed across multiple organ systems in SLE patients (8.4–21.1%). We estimated a larger number of prevalent SLE cases in the US civilian population than previous reports and observed extensive disease burden based on a 1-year cross-sectional analysis.

**Supplementary Information:**

The online version contains supplementary material available at 10.1186/s13104-021-05877-1.

## Introduction

Systemic lupus erythematosus (SLE) is a complex autoimmune disease that can affect multiple body organs and systems [1]. In the US, older prevalence estimates range from 24 to 150 per 100,000 among adults, with a more recent meta-analysis of US registries suggesting a prevalence of 72.8 per 100,000 person-years (95% confidence interval [95% CI] 65.3–81.0) [2, 3]. The recent meta-analysis applied older age-, sex-, and race-specific rates of SLE from registry data collected before 2010 to US population Census data in 2018 to estimate that 204,295 persons (95% CI 160,902–261,725) in the US fulfilled the criteria for SLE [3]. This count is similar to older estimates of 161,000 with definite SLE and 322,000 patients with definite/probable SLE that were calculated by projecting SLE rates in 15–64 year old adults in the San Francisco Kaiser Permanente Health Plan from 1965 to 1973 to the 2005 US Census population data [4]. It is unknown whether prevalence estimates using older, geographically-specific data apply to the current US population since the SLE classification has changed over time [1].

Health insurance claims are a potential, contemporary data source for estimating the proportion and number of prevalent SLE patients in the US and can provide timely, relevant, and geographically-diverse data to support healthcare planning for the disease. In the US, private, Medicare, and Medicaid insurance make up the major civilian health insurance sectors [5]. Prior studies have used insurance data for these estimates, with a recent study finding 97,590 people with prevalent SLE among the total Medicare population in 2016 [6]. Another poster abstract using private, Medicare and Medicaid insurance claims databases estimated a total of 313,436 prevalent SLE patients in the US in 2009 [7]. However, no current estimates of prevalent SLE patients across types of insurance coverage are available in the public domain.

In this report, we provide current estimates of the prevalence proportions and counts of SLE patients in the US by major health insurance types. In addition, we describe 1-year cross-sectional SLE healthcare utilization and encounters across different insurance types.

## Main text

### Data sources

We used four large US health insurance claims databases converted to the OMOP Common Data Model [8]: IBM MarketScan® Commercial (CCAE) [9], IBM MarketScan® Medicare Supplemental (MDCR) [10], Optum De-identified Clinformatics® Data Mart Databases (Optum) [11], and IBM MarketScan® Multi-state Medicaid (MDCD) [12]. CCAE includes both health insurance claims (e.g., inpatient, outpatient, and outpatient pharmacy) and enrollment data from large employers and health plans who provide private healthcare coverage to employees, their spouses, and dependents. MDCR is an administrative health claims database for Medicare-eligible active and retired employees and their Medicare-eligible dependents from employer-sponsored supplemental plans. In MDCR, persons aged < 65 years who had Medicare coverage due to disability, including SLE, were excluded in projections because they were not considered representative of the general US population. Optum is an adjudicated administrative health claims database for members with private health insurance. The population is primarily representative of US commercial claims patients (0–65 years old) with some Medicare patients (65+ years old). MDCD is an administrative health claims database with the pooled healthcare experience of Medicaid enrollees covered under fee-for-service and managed care plans from multiple states.

The use of Optum and MarketScan® databases was reviewed by the New England Institutional Review Board and was determined to be exempt from broad IRB approval, as this research project did not involve human subject research.

### Patients

In each database, prevalent SLE cases were identified for calendar year 2016 based on having ≥ 1 SLE diagnosis codes or ≥ 1 belimumab prescription in 2016 and based on meeting at least one of the following conditions before or during 2016: (1) ≥ 3 SLE diagnoses spanning across ≥ 60 days; or (2) ≥ 1 belimumab infusion/injection and ≥ 2 SLE diagnoses; or (3) ≥ 1 inpatient SLE diagnosis and ≥ 1 dispensed prescription for systemic corticosteroids, antimalarials, or immunomodulators commonly used in SLE treatment [13–17]. Codes used to define conditions and drugs are provided in the Concept definition tables in Additional file 1. At least 1 year of continuous enrollment in the health plan was required. The study flowchart and claims database algorithms for defining SLE based on the published literature are in Additional file 1.

### Analysis

In each database, an age- and sex-specific SLE prevalence proportion was estimated for calendar year 2016. The denominator included individuals enrolled in insurance plans for the entire year of 2016, and the numerator included the subset diagnosed with SLE. Projection of prevalent SLE cases (rounded down to the nearest thousands) in the US was based on the age- and sex-specific prevalence proportions from each database multiplied by the corresponding US census population counts by age, sex, and insurance type, and the sum was taken [18]. Given that CCAE mostly includes patients aged < 65 and MDCR mostly includes patients aged ≥ 65, we combined the estimated SLE prevalence proportions from MDCR age ≥ 65 and CCAE age < 65 to project to a US civilian population with private insurance coverage of all ages. The estimated SLE prevalence proportions from Optum were used as a second source to project to US civilian population with private/Medicare insurance coverage across all ages. These two estimates for people with private/Medicare insurance coverage were combined with projections to persons with Medicaid coverage to estimate overall SLE prevalence in the US civilian population. To enable comparison with age-standardized SLE prevalence previously published in population-based SLE surveillance programs, we provided age-standardized SLE prevalence (Additional file 1: Table S2).

To provide a 1-year, cross-sectional profile of disease burden, we described the healthcare utilization and encounters for various SLE-related conditions and comorbidities among the prevalent SLE population in 2016. We summarized the median (IQR) number of encounters, the number of patients with ≥ 1 hospitalization, and the median (IQR) length of hospitalizations during the year. Additionally, we summarized the number and percentage of patients with ≥ 1 qualifying diagnosis code for comorbidities relevant to SLE and with ≥ 1 medication code for antimalarials, disease-modifying antirheumatic drugs (DMARDs), systemic corticosteroids, or biologics in the insurance claims. Comorbidity and medication codes are provided in the Additional file 1.

### Results

In 2016, a total of 28,848 (CCAE), 3922 (MDCR age ≥ 65), 23,877 (Optum) and 15,096 (MDCD) prevalent cases of SLE were identified. The unadjusted SLE prevalence per 100,000 persons was 150.1 (25.6 in males, 266.0 in females), 236.4 (66.1 in males, 372.7 in females), 195.4 (39.2 in males, 341.1 in females) and 158.7 (27.1 in males, 258.9 in females) in CCAE, MDCR age ≥ 65, Optum, and MDCD, respectively. Age-standardized SLE prevalence per 100,000 persons was 134.9, 146.7, 143.3, and 244.3 in CCAE, MDCR age ≥ 65, Optum, and MDCD, respectively (see Additional file 1).

**Fig. 1:**
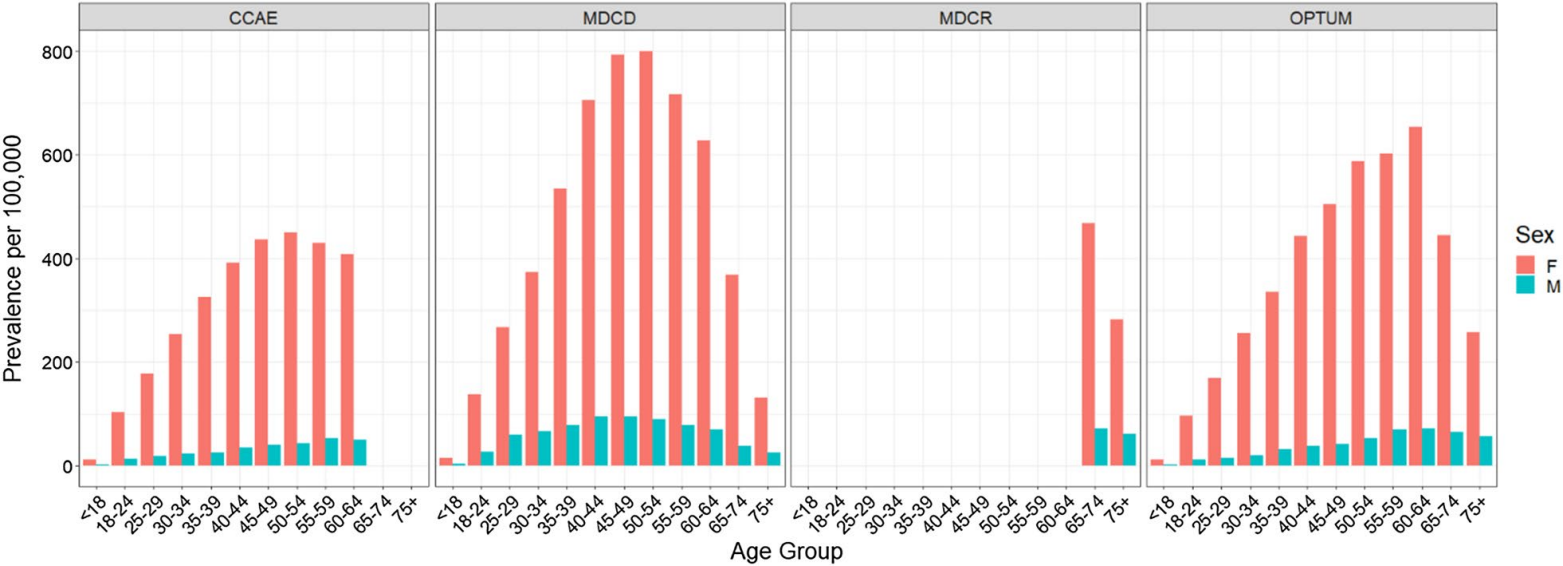
Age-, and sex-specific prevalence of SLE in the 4 study databases, 2016. See Additional file 1: Fig. S1 for prevalence proportions under age 65 for MDCR

The age- and sex-specific prevalence proportions from each database are plotted in Fig. 1. SLE prevalence in females was consistently higher than in males across all age groups in all four databases. The female-to-male ratio of SLE prevalence by age is provided in Additional file 1: Fig. S2. The projected numbers of prevalent SLE cases with private insurance (including Medicare supplemental) or Medicaid insurance ranged from 444,000 (i.e., 345,000 with private/Medicare plus 99,000 with Medicaid insurance) to 503,000 (404,000 with private/Medicare plus 99,000 with Medicaid insurance) (Table 1).

**Table 1:** Estimated total number of prevalent SLE patients in the US, by major civilian health insurance, 2016

Age categories	Projection to private insurance population		Projection to Medicaid population
	CCAE (age < 65) and MDCR (age ≥ 65)^a^	Optum (all ages)^b^	MDCD^c^
< 18			
Female	2465	2214	2148
Male	357	329	426
18–64			
Female	259,502	311,904	80,425
Male	23,067	29,975	6367
≥ 65			
Female	52,784	53,125	8994
Male	7501	6799	524
Total (rounding)	345,000	404,000	99,000

^a^Based on estimated age, sex-specific prevalence proportion from CCAE (age < 65) and MDCR (age ≥ 65) multiplied by US census age and sex-specific counts of persons with private insurance in 2016; age categories were < 18, 18–24, 25–29, 30–34, 35–39, 40–44, 45–49, 50–54, 55–59, 60–64 in CCAE and 65–74, ≥ 75 in MDCR

^b^Based on estimated age, sex-specific prevalence proportion from Optum multiplied by US census age and sex-specific counts of persons with private insurance in 2016; age categories were < 18, 18–24, 25–29, 30–34, 35–39, 40–44, 45–49, 50–54, 55–59, 60–64, 65–74, ≥ 75 in Optum (separately done in those age < 65 and age ≥ 65)

^c^Based on estimated age, sex-specific prevalence proportion from MDCD multiplied by US census age and sex-specific counts of persons with Medicaid insurance in 2016; age categories were < 18, 18–24, 25–29, 30–34, 35–39, 40–44, 45–49, 50–54, 55–59, 60–64, 65–74, ≥ 75 in MDCD

Across the four databases, 15% to 33% of patients had at least one hospitalization in 2016 (Table 2). Healthcare utilization, including hospitalizations for infections, was highest in MDCD despite the database having the lowest mean age. Comorbidities and SLE disease manifestations were common across multiple organ systems in SLE patients, including renal failure/dialysis that ranged from 8.4% (CCAE) to 21.1% (MDCR). Across the four databases, SLE medications dispensed the most frequently were systemic corticosteroids (61–65%) and anti-malarial drugs (35–63%); anti-inflammatory biologic agents were infrequently prescribed (< 5%) in any of the database cohorts.

**Table 2:** Characterization of prevalent SLE patients and their healthcare utilization across 4 US databases, 2016

	CCAE	MDCR	OPTUM	MDCD
Total N	28,846	4281	23,877	15,096
Women, n (%)	26,476 (91.8)	3759 (87.8)	21,568 (90.3)	13,979 (92.6)
Mean age (SD), years	47 (12)	72 (7)	56 (15)	45 (15)
Number of visits, median (interquartile range)				
For any healthcare encounter	17 (10–30)	25 (15–41)	20 (11–35)	25 (13–49)
With an SLE diagnosis	4 (2–7)	3 (2–5)	4 (2–7)	4 (2–8)
Number of patients with ≥ 1 hospitalization, n (%)	4374 (15.2)	1185 (27.7)	4510 (18.9)	5044 (33.4)
Median (interquartile range) length of hospital-stay, days	3 (2–6)	4 (2–8)	4 (2–6)	4 (2–6)
Co-morbidities^a^, n (%)				
Renal diseases	5018 (17.4)	1177 (27.5)	6089 (25.5)	4328 (28.7)
Renal dialysis/failure	2429 (8.4)	902 (21.1)	3970 (16.6)	2781 (18.4)
Cardiovascular diseases				
Hypertension	10,191 (35.3)	2999 (70.1)	11,457 (48.0)	5218 (34.6)
Ischemic heart disease	1031 (3.6)	545 (12.7)	2186 (9.2)	1433 (9.5)
Heart failure	903 (3.1)	638 (14.9)	2275 (9.5)	2047 (13.6)
Rheumatic heart disease	483 (1.7)	236 (5.5)	799 (3.4)	537 (3.6)
Cerebral vascular diseases	1052 (3.7)	484 (11.3)	1747 (7.3)	1409 (9.3)
Neuropsychiatric conditions				
Headache (recorded on claims)	4043 (14.0)	512 (12.0)	3804 (15.9)	3897 (25.8)
Psychosis	444 (1.5)	272 (6.4)	990 (4.2)	1004 (6.7)
Epilepsy/seizure	1050 (3.6)	144 (3.4)	1336 (5.6)	1800 (11.9)
Depression	4112 (14.3)	698 (16.3)	4782 (20.0)	3284 (21.8)
Cutaneous manifestations				
Cutaneous lupus	4452 (15.4)	683 (16.0)	4133 (17.3)	3136 (20.8)
Dermatosis and dermatitis	3977 (13.8)	683 (16.0)	3412 (14.3)	2184 (14.5)
Infections	8778 (30.4)	1450 (33.9)	7955 (33.3)	6405 (42.4)
Hospitalized infections	1543 (5.4)	472 (11.0)	1847 (7.7)	2168 (14.4)
Musculoskeletal comorbidities				
Inflammatory Polyarthropathies	6711 (23.3)	1238 (28.9)	7403 (31.0)	3688 (24.4)
Spondylopathies	3050 (10.6)	874 (20.4)	3999 (16.8)	2337 (15.5)
Osteoarthritis	7257 (25.2)	2251 (52.6)	9360 (39.2)	5145 (34.1)
Osteoporosis	2351 (8.2)	1056 (24.7)	4051 (17.0)	1257 (8.3)
Medication^b^ use (any), n (%)				
Anti-malarials	18,129 (62.8)	2275 (53.1)	12,411 (52.0)	5324 (35.3)
Systemic corticosteroids	18,518 (64.2)	2768 (64.7)	14,619 (61.2)	9255 (61.3)
Non-biologic disease modifying drugs	5906 (20.5)	660 (15.4)	3904 (16.4)	2423 (16.1)
Biologics	1286 (4.5)	139 (3.2)	818 (3.4)	480 (3.2)
Any of the above	25,037 (86.8)	3620 (84.6)	19,268 (80.7)	10,746 (71.2)

Physician specialty is incompletely captured or unspecified in insurance claims databases, especially MDCD

^a^Based on diagnosis codes, except for depression which includes anti-depressant prescriptions (codes provided in Additional file 1)

^b^Antimalarials included artemether, lumefantrine, atovaquone, proguanil, chloroquine, halofantrine, hydroxychloroquine, mefloquine, primaquine, pyrimethamine, quinacrine, quinine, sulfadoxine, pyrimethamine, chloroquine; non-biologic disease modifying drugs included azathioprine, chlorambucil, cyclophosphamide, cyclosporine, methotrexate, mycophenolate mofetil, mycophenolic acid; Biologics included abatacept, rituximab, tocilizumab, adalimumab, etanercept, infliximab, golimumab, certolizumab pegol, ustekinumab, secukinumab, ixekizumab, vedolizumab, belimumab

### Discussion

Our study characterized patient cohorts from four large US insurance claims databases to provide updated estimates of SLE prevalence proportions and the number of SLE cases in the US civilian population. We estimated roughly 345,000 to 404,000 prevalent SLE patients with private/Medicare insurance, and 99,000 prevalent SLE patients with Medicaid insurance in 2016, giving an overall unadjusted SLE prevalence in the US ranging from 150.1 (private insurance beneficiaries aged < 65 years) to 252.9 (Medicare supplemental beneficiaries aged ≥ 65 years). The cross-sectional design also demonstrated considerable comorbidity and medication utilization across all insurance types in 2016.

Our prevalence estimate of SLE in the US is higher than previously reported, including a recent meta-analysis which estimated 204,295 SLE cases (95% CI 160,902–261,725) in the US by applying prevalence estimates from 5 registries in 2002–2004 and 2007–2009 to the 2018 US population [3] and an abstract using claims databases in 2009 [7]. The higher estimates may reflect both increased SLE prevalence proportions and an overall increased US population size. Rigorous algorithms were used to identify SLE cases [13–17], with an estimated sensitivity and specificity both > 90% and positive predictive value 80–90% (Additional file 1: Tables S1, S3). Although chart validation was not feasible, the SLE algorithms reflect the real-world healthcare experience of presumed SLE patients in routine clinical practice. One case definition within the algorithm paired an SLE diagnosis code with various SLE treatments, including a broad list of systemic corticosteroids that may not be used to specifically treat the disease and could introduce misclassified cases. However, the impact of this sensitive definition is likely minimal given that 98–99% of cases across the four databases met the case definition of ≥ 3 SLE diagnoses at least 60 days apart.

### Conclusions

Overall, our research provides an update on the estimates of prevalent cases of SLE from a variety of large US insurance types, each indicating a significant utilization burden. Our study suggests extensive healthcare utilization by SLE cases across the individual insurance types in 2016, especially for Medicaid beneficiaries. Future research is needed to understand the healthcare and societal costs for the management of persons diagnosed with SLE, including causes of health disparities, resulting disability, and premature mortality.

## Limitations

Some limitations should be considered when interpreting these findings. First, the four databases do not cover the 27.5 million US persons without health insurance in 2016 and may not include all US persons with health insurance. Our prevalence estimates may be an underestimate or overestimate of the true SLE prevalence in the US if the missing populations have a different proportion of prevalent SLE than the study population. Also, only 50% of the US population covered by Medicare insurance have the supplemental Medicare insurance used in our study. However, a prior study using a 20% random sample of Medicare data estimated similar numbers of patients with SLE (n = 54,490) as we found in the Medicare supplemental data (n = 60,285) [6].

Our estimate of prevalent SLE cases was higher than in older studies, which may be explained by our prevalence definition. Li and colleagues [6] defined prevalent SLE by requiring all the SLE criteria to be met in the cohort year, whereas our study defined prevalent SLE by requiring all SLE criteria to be met during or before 2016. Due to flare/remission disease patterns of SLE, [14] our more sensitive definition may capture prevalent SLE cases who happened to be inactive or in remission in 2016 and not included by Li et al. [6].

Another study limitation is that we cannot describe the SLE burden by race/ethnicity since this information is only available in the MDCD database. MDCD has a distribution of race/ethnicity that is very different than the distribution in the US population, including more than double the proportion of Black patients. Using race/ethnicity results from these data would provide skewed information on the burden of SLE by race/ethnicity in the US.

Finally, we could not account for persons with multiple insurance types who are found in more than one health insurance database. Adding the projected SLE cases across different insurance types may overestimate the total number of SLE cases in the US. For example, Li and colleagues reported about 13% of SLE patients had dual Medicare and Medicaid coverage [6]. However, we expect overlap between Medicare and Medicaid in CCAE and Optum to be low given that they only include patients with Medicare supplemental coverage.

## Supplementary Information

**Additional file 1.** Table S1.

## Data Availability

The data underlying this article were made available to the authors by third-party license from IBM MarketScan®, and Optum®, commercial data providers in the US. Under the licensing agreement, the authors cannot provide the raw data themselves. Other researchers could access the data by purchase through IBM MarketScan® and Optum®; and the inclusion criteria specified in the Methods section would allow them to identify the same cohort of patients we used for these analyses.

## Abbreviations

SLE: Systemic lupus erythematosus
US: United States
CCAE: IBM MarketScan® Commercial database
MDCR: IBM MarketScan® Medicare Supplemental database
Optum: Optum De-identified Clinformatics® Data Mart Databases
MDCD: IBM MarketScan® Multi-state Medicaid database

## Code availability

The common data model (CDM) methods are openly available from https://www.ohdsi.org/data-standardization/the-common-data-model/. Specific diagnosis and drug codes in the study are provided in Additional file 1.
