# Supplementary material for: Update on prevalence of diagnosed systemic lupus erythematosus (SLE) by major health insurance types in the US in 2016
Source: BMC Res Notes. 2022 Jan 9;15:5. doi: 10.1186/s13104-021-05877-1 (PMC8744244; doi:10.1186/s13104-021-05877-1)
Supplement: Supplementary file 1 — Additional file 1. Table S1. [file 13104_2021_5877_MOESM1_ESM.docx]

Additional Materials to **Prevalence of Diagnosed Systemic Lupus Erythematosus (SLE), and Patient Characteristics by Major Health Insurance Types in the US**

**Contents**

[Concept Definitions 2](#_Toc87036703)

[Concept sets for SLE algorithm 2](#_Toc87036704)

[Concept sets for kidney disease or related procedures 2](#_Toc87036705)

[Concept sets for cardiovascular conditions 3](#_Toc87036706)

[Concept sets for neuropsychiatric disorders 4](#_Toc87036707)

[Concept sets for cutaneous manifestations 5](#_Toc87036708)

[Infections incl. opportunistic infections (non-vector, non-STD, non-congenital, not in pregnancy) 5](#_Toc87036709)

[Musculoskeletal comorbidities 5](#_Toc87036710)

[Additional tables 6](#_Toc87036711)

[Table S1. Study cohort creation flow, 2016. 6](#_Toc87036712)

[Table S2. Summary of prevalence estimates from registries published in the literature versus the current study 7](#_Toc87036713)

[Table S3. Summary of SLE algorithms in the literature 9](#_Toc87036714)

[Additional Figures 14](#_Toc87036715)

[Additional Figure SF1. Age-, sex-specific SLE prevalence, 2016 14](#_Toc87036716)

[Additional Figure SF2. Female: Male ratio of SLE prevalence by age, 2016 15](#_Toc87036717)

[Additional References 16](#_Toc87036718)

# Concept Definitions

### Concept sets for SLE algorithm

Patients defined as having SLE if they meet one of the following three case definitions: (1) *≥3 SLE dx (ICD-9 710.0 or ICD-10 M32.10)******** *spanning at least 60 days, or (2) ≥1 belimumab prescription and >=1 SLE dx, or (3) ≥1 inpatient dx of SLE (ICD-9 710.0 or ICD-10 M32.10), and ≥1 Rx (systemic corticosteroids, antimalarials, or Immunomodulators. Codes for these definitions are provided below.*

| **Concept** | **Standard Codes** | **Notes** |
| --- | --- | --- |
| [Systemic Lupus Erythematosus](https://epi.jnj.com/atlas/#/conceptset/7199/conceptset-expression) |  | Removed drug-induced lupus |
| [Belimumab](https://epi.jnj.com/atlas/#/conceptset/7168/conceptset-expression) |  | Ingredient-level belimumab |
| [Systemic Corticosteroids](https://epi.jnj.com/atlas/#/conceptset/7073/conceptset-expression) |  | Added the following ingredients: *betamethasone, budesonide, cortisone, desoxycorticosterone, dexamethasone, fludrocortisone, hydrocortisone, methylprednisolone, paramethasone, prednisolone, prednisone, triamcinolone*  AND  Removed concepts under the following classifications: *vaginal product, topical product, toothpaste product, shampoo product, rectal product, otic product, opthamalic product, nasal product, medicated pad/tape, lozenge product, inhalant product* |
| [Antimalarials](https://epi.jnj.com/atlas/#/conceptset/7166/details) |  | Added the following ingredients:  *Artemether, lumefantrine, atovaquone, proguanil, chloroquine, halofantrine, hydroxychloroquine, mefloquine, primaquine, pyrimethamine, quinacrine, quinine, sulfadoxine, pyrimethamine, chloroquine* |
| [Non-biologic DMARDS](https://epi.jnj.com/atlas/#/conceptset/7828/details) |  | Added the following ingredients:  *Azathioprine, chlorambucil, Cyclophosphamide, Cyclosporine, Methotrexate, mycophenolate mofetil, mycophenolic acid* |
| [Biologics](https://epi.jnj.com/atlas/#/conceptset/7829/included-conceptsets) |  | Added the following ingredients:  *Abatacept, rituximab, tocilizumab, adalimumab, etanercept, infliximab, golimumab, certolizumab pegol, ustekinumab, secukinumab, ixekizumab, vedolizumab, belimumab* |

### Concept sets for kidney disease or related procedures

Patients considered to have a kidney or kidney-related disease If they had one of the following

| **Concept** | **Standard Codes** | **Notes** |
| --- | --- | --- |
| [Kidney diseases](https://epi.jnj.com/atlas/#/conceptset/7864/conceptset-expression) | / | SNOMEDs that map to the following :  **ICD-9 dx**: 580 thru 586, and 791.0, V42.0, V56, 996.81;  **ICD-10 dx**: N00 through N19, and R80, Z94.0, Z49, T86.1 |
| [Kidney biopsy procedures](https://epi.jnj.com/atlas/#/conceptset/7942/conceptset-expression) |  | Used the suggested CPT4 codes and added ICD-10-PCS codes for excision or drainage of the kidney (either side) |
| [Hemodialysis, peritoneal dialysis or kidney transplant, or renal failure](https://epi.jnj.com/atlas/#/conceptset/7965/conceptset-expression) |  | Please see the concept set for CPT4 and ICD-10-PCS codes |

### Concept sets for cardiovascular conditions

| **Concept** | **Standard Codes** | **Notes** |
| --- | --- | --- |
| [Hypertension](https://epi.jnj.com/atlas/#/conceptset/7867/conceptset-expression) |  |  |
| Antihypertensive medications |  | Includes the following ingredients:  [Renin-angiotensin system blockers](https://epi.jnj.com/atlas/#/conceptset/7868/conceptset-expression) (i.e., ACEs and ARBs): ALISKIREN BENAZEPRIL CANDESARTAN CAPTOPRIL CILAZAPRIL ENALAPRIL EPROSARTAN FOSINOPRIL IMIDAPRIL IRBESARTAN LISINOPRIL LOSARTAN MOEXIPRIL OLMESARTAN PERINDOPRIL QUINAPRIL RAMIPRIL SPIRAPRIL TELMISARTAN TRANDOLAPRIL VALSARTAN SPIRAPRIL ZOFENOPRIL Verapamil;  [Thiazides](https://epi.jnj.com/atlas/#/conceptset/7901/conceptset-expression): BENDROFLUMETHIAZIDE CHLOROTHIAZIDE CHLORTHALIDONE HYDROCHLOROTHIAZIDE HYDROFLUMETHIAZIDE INDAPAMIDE METHYCLOTHIAZIDE METIPAMID METOLAZONE POLYTHIAZIDE TRICHLORMETHIAZIDE;  [Loop diuretics](https://epi.jnj.com/atlas/#/conceptset/7902/conceptset-expression): BUMETANIDE FUROSEMIDE PIRETANIDE TORSEMIDE;  [Potassium sparing](https://epi.jnj.com/atlas/#/conceptset/7903/conceptset-expression): AMILORIDE TRIAMTERENE;  [Aldosterone receptor blocker](https://epi.jnj.com/atlas/#/conceptset/7904/conceptset-expression): EPLERENONE SPIRONOLACTONE;  [Other diuretics:](https://epi.jnj.com/atlas/#/conceptset/7905/conceptset-expression) ACETAZOLAMIDE ALDACTAZIDE CAPOZIDE CLOPAMIDE DICHLORPHENAMIDE DYAZIDE DYNORM EPLERENONE ETACRYNIC METHAZOLAMIDE  SPIRONOLACTONE XIPAMIDE;  [Beta-blockers:](https://epi.jnj.com/atlas/#/conceptset/7906/conceptset-expression) ACEBUTOLOL ALPRENOLOL ATENOLOL BETA-ADRENERGIC BETAXOLOL BISOPROLOL BISELECT CARVEDILOL CELIPROLOL LABETALOL METIPRANOLOL METOPROLOL MODILOC NADOLOL NEBIVOLOL OXPRENOLOL PENBUTOLOL PINDOLOL PROPRANOLOL SELOKEN SOTALOL TIMOLOL ALPRENOLOL CELIPROLOL METIPRANOLOL OXPRENOLOL TENORETIC;  [Calcium channel blockers](https://epi.jnj.com/atlas/#/conceptset/7907/conceptset-expression): AMLODIPINE DILTIAZEM FELODIPINE ISRADIPINE NICARDIPINE NIFEDIPINE NISOLDIPINE VERAPAMIL NIMODIPINE;  [Alpha receptor blockers](https://epi.jnj.com/atlas/#/conceptset/7909/conceptset-expression): DOXAZOSIN PRAZOSIN TERAZOSIN;  [Other hypertension meds](https://epi.jnj.com/atlas/#/conceptset/7910/conceptset-expression): ALFUZOSIN CLONIDINE DESERPIDINE DOXAZOSIN GUANABENZ GUANADREL GUANETHIDINE GUANFACINE HYDRALAZINE METHYLDOPA MINOXIDIL PRAZOSIN RESERPINE SILODOSIN TAMSULOSIN TERAZOSIN |
| [Ischemic heart disease](https://epi.jnj.com/atlas/#/conceptset/7911/conceptset-expression) |  | SNOMEDs mapping to:  (ICD-9 410-414; ICD-10 I20-I25), |
| [Heart failure](https://epi.jnj.com/atlas/#/conceptset/7912/conceptset-expression) |  | SNOMEDs mapping to: (ICD-10 I50), |
| [Chronic rheumatic heart disease](https://epi.jnj.com/atlas/#/conceptset/7912/conceptset-expression) |  | SNOMEDs mapping to: (ICD-10 I05-I09), |
| [Cerebrovascular diseases](https://epi.jnj.com/atlas/#/conceptset/7915/conceptset-expression) |  | SNOMEDs mapping to:  ICD-10 I60-I69 plus ICD-10 G43.6 or G46 |

### Concept sets for neuropsychiatric disorders

| [Headache](https://epi.jnj.com/atlas/#/conceptset/7917/conceptset-expression) |  | SNOMEDs mapped to icd-9 diagnosis 784.0, icd-10 R51  Note: Cannot remove G44.5 and G44.8 if we use mapping to SNOMED. |
| --- | --- | --- |
| [Epilepsy/Seizures](https://epi.jnj.com/atlas/#/conceptset/7918/conceptset-expression) |  | SNOMEDs mapping to:  icd-9 diagnosis 345, 780.39, icd-10 G40, R56.9 |
| [Psychosis](https://epi.jnj.com/atlas/#/conceptset/7919/conceptset-expression) |  | SNOMEDs mapping to: (icd-9 290, 294, 295-299, icd-10 F01-F09, F20-F29), |
| [Depression](https://epi.jnj.com/atlas/#/conceptset/5894/conceptset-expression) |  |  |
| Anti-depressants | See links to Atlas concept sets in the right-hand column | Includes the following ingredients:  [**SSRIs:**](https://epi.jnj.com/atlas/#/conceptset/7920/conceptset-expression) CITALOPRAM ESCITALOPRAM FLUOXETINE FLUVOXAMINE PAROXETINE SERTRALINE;  [**SNRI**](https://epi.jnj.com/atlas/#/conceptset/7921/conceptset-expression)**s**: DESVENLAFAXINE DULOXETINE MILNACIPRAN VENLAFAXINE levomilnacipran;  [**TCAs**](https://epi.jnj.com/atlas/#/conceptset/7922/conceptset-expression)**:** AMITRIPTYLINE AMOXAPINE CLOMIPRAMINE DESIPRAMINE DOXEPIN IMIPRAMINE NORTRIPTYLINE PROTRIPTYLINE SEROTONIN TRIMIPRAMINE;  [**MAOIs:**](https://epi.jnj.com/atlas/#/conceptset/7923/conceptset-expression) ISOCARBOXAZID PHENELZINE TRANYLCYPROMINE; *note: SELEGILINE is mainly used to treat early Parkinson’s disease, so not included.  [**NAs**](https://epi.jnj.com/atlas/#/conceptset/7924/conceptset-expression)**:** BUPROPION VILAZODONE MAPROTILINE MIRTAZAPINE NEFAZODONE TRAZODONE; |

### Concept sets for cutaneous manifestations

| [Cutaneous lupus](https://epi.jnj.com/atlas/#/conceptset/7925/conceptset-expression) |  | SNOMEDs mapping to ICD-9 695.4; ICD-10 L93.0 thru L93.2  Notes: Relied on ICD-10 codes since only looking in 2017. Added H01.121, H01.122. H01.123, H01.124, H01.125, H01.126, H01.129 (discoid lupus of the eyelids) |
| --- | --- | --- |
| [Dermatosis/dermatitis](https://epi.jnj.com/atlas/#/conceptset/7926/conceptset-expression) |  |  |

### Infections incl. opportunistic infections (non-vector, non-STD, non-congenital, not in pregnancy)

| [Infections](https://epi.jnj.com/atlas/#/conceptset/7962/included-sourcecodes) |  | Very large list. Please see excel spreadsheet for SNOMED and the ICD-10CM/ICD-9CM that they map to. They do not 100% map to the recommended list provided but I tried to remove any vector-borne diseases, STDs (beyond HCV and HBV) and congenital/natal infections as seemed to be what was done in the provided list. |
| --- | --- | --- |

### Musculoskeletal comorbidities

| [Inflammatory polyarthopathies](https://epi.jnj.com/atlas/#/conceptset/7929/included-conceptsets) |  | SNOMED codes mapping to ICD-10 M05-M14  Notes: Also including ICD-10 M1A for chronic gout since we are including M10 (cannot separate the two)t. Removed M08.1 for juvenile ankylosing spondylitis since we don’t include other ankylosing spondylitis (M45). |
| --- | --- | --- |
| [Spondylopathies](https://epi.jnj.com/atlas/#/conceptset/7934/conceptset-expression) |  | SNOMED codes mapping to ICD-10 M45-M49  Notes: M08.08, M08.1, M08.28 and M12.18 added here and removed from polyarthropathies. |
| [Osteoarthritis](https://epi.jnj.com/atlas/#/conceptset/7937/conceptset-expression) |  | SNOMED codes mapping to ICD-10 M15-M19 |
| [Osteoporosis](https://epi.jnj.com/atlas/#/conceptset/7937/included-conceptsets) |  | SNOMED codes mapping to ICD-10 M80-M85  Notes: Not including M85.2 hypertosis of the skull, M85.8 or M85.9. |

# Additional tables

## Table S1. Study cohort creation flow, 2016.

| Inclusion/ Exclusion cascade | IBM CCAE | IBM MDCR** | Optum SES | IBM MDCD |
| --- | --- | --- | --- | --- |
| ≥1 SLE dx or ≥1 belimumab infusion/injection between 1/1/2016-12/31/2016 | 44010 (100%) | 5980 (100%) | 39100 (100%) | 22400 (100%) |
| Continuous enrollment for ≥1 year | 41815 (95%) | 5837 (98%) | 36176 (93%) | 20205 (90%) |
| Patients with: (1) ≥1 SLE dx or ≥1 belimumab infusion/injection between 1/1/2016-12/31/2016, and (2) with continuous enrollment for ≥1 year, and (3) that meet one of the algorithm criteria below before the end of 2016 | 32963 (75%) | 4699 (79%) | 28397 (73%) | 16362 (73%) |
| - *≥3 SLE dx (ICD-9 710.0 or ICD-10 M32.10)* spanning at least 60 days, or* | *32681* | *4608* | *28136* | *15963* |
| - *≥1 belimumab prescription and >=1 SLE dx, or* | *462* | *30* | *162* | *623* |
| - *≥1 inpatient dx of SLE (ICD-9 710.0 or ICD-10 M32.10), and ≥1 Rx (systemic corticosteroids, antimalarials, or IMM)* | *4867* | *1165* | *4627* | *7689* |
| Patients with: (1) ≥1 SLE dx or ≥1 belimumab infusion/injection between 1/1/2016-12/31/2016, (2) with continuous enrollment for ≥1 year, (3) that meet one of the algorithm criteria below before the end of 2016, and (4) continuous enrollment covers entire 2016 | 28848 (66%) | 4281 (72%)* | 23877 (61%) | 15096 (67%) |
| - *≥3 SLE dx (ICD-9 710.0 or ICD-10 M32.10) spanning at least 60 days, or* | *28633*  *(99.3% of 28848)* | *4216 (98.5% of 4281)* | *23695 (99.2% of 23877)* | *14747 (97.7% of 15096)* |
| - *≥1 belimumab prescription and >=1 SLE dx, or* | *288*  *(1.0% of 28848)* | *27 (0.6% of 4216)* | *96 (0.4% of 23877)* | *579 (3.8% of 15096)* |
| - *≥1 inpatient dx of SLE (ICD-9 710.0 or ICD-10 M32.10), and ≥1 Rx (systemic corticosteroids, antimalarials, or IMM)* | *542 (13.7% of 3954)* | *1005 (23.5% of 4216)* | *3766 (15.8% of 23877)* | *7057(46.7% of 15096)* |

*The code for drug-induced systemic lupus erythematosus (ICD-10-CM M32.0) was not included in the SLE definition in order to remove patients with drug-induced SLE from the case definition. However, there is no corresponding ICD-9-CM code, which indicates that drug-induced SLE cases could have been included as prevalent SLE cases diagnosed before 2016.

**In MDCR, n=3,922 (92%) of the 4281 prevalent SLE cases were age 65 or above, and only these patients were included in analysis that projects to the general US population. The unadjusted SLE prevalence per 100,000 persons was 252.9 (69.1 in males and 401.2 in females) in the overall MDCR population. The prevalence proportion for MDCR age 65 or above is reported in the main text of the manuscript

## Table S2. Summary of prevalence estimates from registries published in the literature versus the current study

| Reference | Data Source | Calendar year of prevalence estimate | Denominator (millions, MM) | Numerator (prevalent SLE cases) | Overall Crude Prevalence (per 100,000) | Age-adjusted* Prevalence (per 100,000) |
| --- | --- | --- | --- | --- | --- | --- |
| Lim, 2014 | Georgia Lupus Registry | 2002 | 1.6 MM residents of 2 counties: Fulton and DeKalb | 1,156 cases by ACR‡ classification (fulfilled ≥ 4 ACR criteria);N=1,446 cases by combined definition | 74.4 (95% CI 70.3–78.9) by ACR classification; 93.1 (95% CI 88.4-98) by combined definition | 73.0 (95% CI 68.9-77.4) by ACR classification; 92.1 (95% CI 87.4-97) by combined definition |
| Somers, 2014 | Michigan Lupus Epidemiology and Surveillance program | 2002 | 2.4 MM residents of 2 counties: Wayne and Wash-tenaw | 2,278 (2,139 by ACR classification) | 72.1 (95% CI 70.1–74.1) | 72.8 (95% CI 70.8–74.8) |
| Dall’Era, 2017 | California Lupus Surveillance Project | 2007 | 0.8 MM residents of San Francisco County, averaged 2007–2009 | 759 by ACR classification | 96.0 (95% CI 89.4–103.1) by ACR classification; 109.9 (95% CI 102.8–117.4) by combined definition | 84.8 (95% CI 78.6–91.5) by ACR classification; 96.8 (95% CI 90.2–103.9) by combined definition |
| Izmirly, 2017 | Manhattan Lupus Surveillance Program | 2007 | 1.6 MM residents in 2010 in New York County (Manhattan) | 1,078 by ACR classification, N=1,267 by SLICC, n=1,256 by treating rheumatologist diagnosis | 68.2 (95% CI 64.1–72.2) by ACR classification; 80.1 (75.7–84.5); 79.4 (95% CI 75.0–83.8) by rheumatologist diagnosis | 62.2 (95% CI 58.4–66.0) by ACR classification; 73.8 (95% CI 69.6–77.9) by SLICC, 72.6 (95% CI 68.5–76.7) by rheumatologist diagnosis |
| Ferucci, 2014 | Indian Health Service Lupus registry | 2007 | 0.2 MM combined (0.1 MM Alaska, 0.07 MM Phoenix, 0.02 MM Oklahoma) | 285 cases (130 Alaska, 125 Phoenix, 30 Oklahoma) | 134 (120–151) by primary definition | 178 (157–200) by primary definition |
| Izmirly, 2021 | Meta-analysis of prevalence estimates from Georgia Lupus Registry; Michigan Lupus Epidemiology and Surveillance program, California Lupus Surveillance Project, Manhattan Lupus Surveillance Program,Indian Health Service Lupus registry) | 2002-2004 (Georgia Lupus Registry and Michigan Lupus Epidemiology and Surveillance program)  2007-2009 (California Lupus Surveillance Project, Manhattan Lupus Surveillance Program, Indian Health Service Lupus registry) | 6.6 MM (Assuming no overlap in the 5 registries) | 5417 cases (meeting ACR classification) | Not reported | 72.8 per 100,000 person-years (95% confidence interval [95% CI] 65.3–81.0) |
| Current study | 4 health insurance databases | 2016 (9 to 14 years later than the CDC-funded studies. | 43 MM combined (19.2 million (MM) in CCAE, 1.7 MM in MDCR , 12.2 MM Optum, 9.5 MM in MDCD | 72,102 cases combined (including 28,848 CCAE; 4,281 MDCR; 23,877 Optum, 15,096 MDCD).SLE definition details provided in Additional Table S3 | 150.1 (95% CI 148.4-151.8) in CCAE;252.9 (95% CI 245.4-260.6) in all MDCR; 195.4 (95% CI 192.9-197.9) in Optum 158.7 (95% CI 156.2-161.3) in MDCD | 134.9 (95% CI 129.7-140.1) CCAE**; 146.7 (140.9-152.9) in MDCR age ≥65 (no counterpart from CDC funded studies of SLE prevalence restricted to this age;143.3 (95% CI 141.2-145.4) Optum; 244.3 (95% CI 240.2-248.3) MDCD |

Abbreviations: ACR, American College of Rheumatology; CDC, U.S. Centers for Disease Control and Prevention; SLICC, Systemic Lupus International Collaborating Clinics classification criteria

*Age standardized to US 2000 population standard (Klein, 2001), <https://seer.cancer.gov/stdpopulations/stdpop.singleages.html>

**Note that age distribution is very different between the US 2000 Standard population and people covered by commercial insurance mostly through employers (CCAE), and people under the age of 65 years who qualify for Medicare as well as commercial supplemental coverage (MDCR). For example, 20.7% versus 0.04% of weight is given to age 45-49 in the US 2000 Standard population vs MDCR. The latter reflects the burden of disability among patients with SLE in the Medicare supplemental population, but age standardization is not appropriate and therefore in the main text of the manuscript, our projection did not include people in MDCR under age 65.

‡ SLE classification criteria exist such as the American College of Rheumatology (ACR) 1997 criteria (Hochberg, 1997; Tan, 1982), the Systemic Lupus International Collaborating Clinics (SLICC) criteria 2012 (Petri, 2012) as well as the new classification criteria jointly supported by the European League Against Rheumatism (EULAR) and ACR (Aringer, 2019a). These criteria are used mainly for identification of relatively homogeneous groups of patients for inclusion in research studies and clinical trials rather than as diagnostic criteria in routine clinical care (Aringer, 2019b; Thong, 2017), although they can be useful to support the initial diagnostic thoughts (Gergianaki, 2018). If a patient meets at least four of the eleven ACR criteria, lupus can be diagnosed with 95% specificity and 85% sensitivity (Lam, 2016). Rheumatologists may not be in perfect agreement on SLE diagnosis (e.g., Bernado, 2017). Even without available “gold-standard”, a trade-off between sensitivity and specificity must be considered for health administrative claims data, and the study objective of understanding SLE prevalence and burden in the different insurance systems.

## Table S3. Summary of SLE algorithms in the literature

| Reference | Study sample and data source | SLE Algorithm | Notes for the present study |
| --- | --- | --- | --- |
| Barnado, 2017  [ref 9 of the present study] | Out of N=5959 patients with at least 1 SLE diagnosis (dx) (ICD-9 710.0) from the Vanderbilt's Synthetic Derivative (SD), a de-identified Electronic Health Records (EHR) database, 200 were randomly selected for chart review | Highest PPV at 95% in the training set (n=100) and 91% in the validation set was ≥3 counts of the SLE ICD-9 code, ANA positive (≥ 1:40), and ever use of both disease-modifying antirheumatic drugs (DMARDs) and steroids, while excluding individuals with systemic sclerosis and dermatomyositis ICD-9 codes. | To evaluate SLE prevalence and burden for health care delivery planning, as in this study, the algorithm should have both high sensitivity and high specificity.  Positive predictive value (PPV) depends on disease prevalence and specificity.  Assuming sensitivity of SLE algorithm=0.9, SLE prevalence 250 per 100,000, PPV of 0.9 implies that the specificity of the algorithm is > 0.99 (Tenny, 2020).  “Use of systemic corticosteroids and other immunomodulators improve the accuracy of SLE identification”, therefore adopted for the present study to combine with the inpatient diagnosis criterion.  Antibody test results are not available in health administrative claims database for the present study, although none of these tests including ANA are perfectly sensitive and specific for SLE.  SLE is a clinical diagnosis, rheumatologists’ diagnosis may be practically the imperfect “gold standard”; citing (Bernado, 2017): “A second rheumatologist (CC) chart reviewed a randomly selected 50 of the 200 charts in the training set. Of the 50 charts, the second rheumatologist's determination of case status was the same as the original rheumatologist (AB) with 96% agreement.” |
| Garris, 2013  [ref 10 of the present study] | N=7,428 patients with SLE identified from the Optum database, 2004-2005, | Age 18–64 years, with criteria  (1) a SLE diagnosis 2004-2005;  (2) during the 36 months after the first SLE claim, ≥3 rheumatologist visits on separate dates with ICD-9-CM SLE dx 710.0x, or ≥2 rheumatologist visits ≥60 days apart with SLE dx, or ≥2 rheumatologist visits on separate dates with SLE dx and ≥1 SLE Rx (oral or IV corticosteroids, anti-malarial, or immunosuppressants);  (3) continuously enrolled for 1 year before and 2 years after first SLE dx. | Optum database in (Garris, 2013) is the same data source of the Optum database in the present study.  In (Garris, 2013), out of 25,182 (100%) patients with at least 1 SLE diagnosis during the study period, only 7,428 (29%) met criteria (2), resulting in an estimated SLE prevalence of 44 per 100,000. This is numerically lower than the estimated 50 per 100,000 from the San Francisco HMO study during 1965-1973 (also lower than estimates from CDC funded SLE surveillance registries, ranging roughly from 70 to 134 per 100,000 in 2002 and 2007).  Across the claims databases in the present study, we found that health care provider specialty is not always available or specified (e.g., “MultiSpecialty Physician Group”, “Medical Doctor (MD), Not elsewhere classified”) on insurance claims, which can result in unnecessary sample size reduction, and not all patients may visit or access specialty care in a 1-year span in 2016. For example, the median (interquartile range) visit to rheumatologist in 2016 was 1 (0-3), 0 (0-2), 1 (0-3) and 0 (0-0) in CCAE (commercial), MDCR (Medicare with employer supplemental coverage), Optum, and MDCD (Medicaid), respectively.  Therefore, we did not impose the specialty criteria for SLE. |
| Kan, 2016  [ref 11 of the present study] | N= 1,611 adults (age≥18) with newly diagnosed SLE, MarketScan® CCAE, 2002-2008 | Modified based on Ref# 29 (Furst, 2013):  1)SLE index date designated as date of first SLE diagnosis (ICD-9 code 710.0);  2) No pre-index SLE dx record (for incident cases);  3) ≥1 inpatient or ≥2 outpatient (at least 30d apart) during 1 year post-index;  4) ≥1 SLE diagnosis at index or during 2 year post index made by a rheumatologist, dermatologist,  nephrologist, or neurologist. | 1) We used the same ICD-9 codes and the corresponding ICD-10-CM codes;  2) we studied prevalent cases and therefore not needed;  3) and 4) were slightly modified, not restricted to specialty (see above), on the other hand, we added prescription records of common SLE therapies as additional evidence to reduce perceived specificity, which is also consistent with our objective of understanding SLE health care burden to different insurance systems.  Requirement of “continuous medical and pharmacy benefits for 12 months before index and 48 months after Index” by (Kan, 2016) is unnecessarily restricted for the present study, and surviving 60 months can exclude SLE deaths. |
| Ke, 2015  [ref 12 of the present study] | N=155 patients who used Belimumab from the HealthCore Integrated Research Database, 2011-2012 | Patients with at least 1 insurance claim for belimumab 2011-2012,  ≥6 months pre-and ≥6 months post-index continuous medical and pharmacy eligibility, and at least 1 medical claim for SLE during the 6-month pre-index period or 6-month post-index period. | (Ke, 2015) reported that all of the belimumab users had an SLE diagnosis on at least 1 medical claim. This is the only biologic agent that has been approved for SLE indication in the US. We adopted this algorithm in the present study for SLE identification because of its high specificity. |
| Moores, 2013 [ref 13 of the present study] | This is a review article for various SLE algorithms from diverse data sources. | Reported 12 algorithms with a validation in the US and Canada, data sources included medical charts and pregnancy exposure programs, as well as the CDC funded SLE surveillance programs. In addition, reported database algorithms without a validation study (published prior to 2013). | Not all sources reported validation performance measures of sensitivity, specificity and PPV.  “Gold” standard included chart review and physician assessment, as well as ACR 1982 and 1997 criteria.  Andrade (2012) [ref#16 of (Moores, 2013)] in the US, used ICD-9-CM 710.0 on ≥2 outpatient visits at least 30 days apart or ≥1 inpatient visit with a diagnosis code during the 1 year prior to pregnancy through date of delivery, using the Medication Exposure in Pregnancy Risk Evaluation Program (MEPREP) database, comprising data from 11 affiliated health plans in the US. Through chart review, PPV (95% CI) calculated based on available data from all encounters was 93% (85–97%).  Assuming PPV of 0.9, sensitivity of 0.9, with SLE prevalence of 250 per 100,000, we estimated the specificity to be >0.99 (Tenny, 2020).  On the other hand, a study using Canada health claims database Bernatsky (2011). |
| Gomez-Puerta, 2015 | N= 42,221 prevalent SLE patients, 8,191 had lupus nephritis (LN), Medicaid Analytic eXtract 2000–2006 from 47 U.S. states (Maine, Tennesee, and Arizona did not contribute) and D.C, adults ages 18–65 years | ≥ 3 ICD-9 codes 710.0 at least 30 days apart, from hospital discharge diagnoses or physician visit claims;  LN: ≥ 2 additional ICD-9 hospital discharge diagnoses or physician billing claims for nephritis, proteinuria and/or renal failure, on or after the date of SLE diagnosis, and occurring at least 30 days apart | Citing Ref #17 (Chibnik, 2010) from (Gomez-Puerta, 2015), positive predictive value (PPV) 80% for LN in Medicaid data.  Because mortality is the primary outcome of (Gomez-Puerta, 2015), deaths were confirmed using the National Death Index, which is not available in the present study. |
| Furst, 2013 | N=1,557 subjects with SLE in the incidence cohort, and 15,396 in the prevalence cohort, Optum 2003-2008 (US) | 1) ≥18 years on service date;  2) continuously enrolled for 24 months before and 12 months after service date;  3) in the 12 months after service date, ≥ 1 inpatient claim or ≥ 2 office or ER visits with an SLE diagnosis;  4) no SLE diagnosis 24 months prior to service date; and  5) no SLE medications 12 months prior to service date | The present study is about prevalence, therefore no restrictions on 4) and 5).  The annual prevalence of SLE (per 100,000 individuals) varied from 81 in 2003 to 103 in 2008 in (Furst, 2013)  Requiring specialist (included rheumatologist, dermatologist, neurologist, or nephrologist) decreased prevalence by about 13% across each year when counting only SLE subjects who had a specialist visit on the prevalence date and/or a specialist visit within a year following the prevalence date.  On the other hand, we imposed ≥3 outpatient (including Emergency Department) visits at least 60 days apart and required prescription evidence in addition to inpatient SLE diagnosis.  We only required 1 year of continuous enrollment for the present study in order to estimate a cross-sectional 1-year SLE burden. |
| Feldman, 2013 | 34,339 individuals with SLE (prevalence = 143.7/100,000) and 7,388 (21.5%) with LN (prevalence = 30.9/100,000) identified from the Medicaid Analytic eXtract data. | SLE identified by> 3 visits with ICD-9 code 710.0 and >30 days apart;  lupus nephritis (LN) by >2 ICD-9 codes for glomerulonephritis, proteinuria or renal failure.  Ages 18-65 years, enrolled in Medicaid for >3 months in 2000-2004 | Our study focused on SLE prevalence and healthcare burden in major insurance types across 1 year.  The (Feldman, 2013) abstract and main text were inconsistent as to whether >3 or >=3 SLE codes were used, we adopted >=3 counts based on majority of studies using the latter; we also adopted visits ≥60 days apart as in (Garris, 2013) because of a common data source using the Optum database, and because of our interest in a relatively more chronic/stable health care utilization. |
| Chibnik, 2010 | N=234 subjects were identified and medical records reviewed from inpatient and outpatient billing data from Medicaid patients at Brigham and Women's Hospital in Boston, 2000-2007 | > 2 ICD-9 codes for SLE 710.0, and (  (a), either > 2 renal ICD-9 codes alone,  Or  (b), > 2 nephrologist visits alone,  Or both (a) and (b) above, or either of (a) or (b)) | PPVs for lupus ranged from 89-92% and PPVs for lupus nephritis ranged from 79-88%, based on the combination of (a) and (b) in addition to >2 ICD-9 codes for SLE 710.0.  (Feldman, 2013) applied this algorithm and cited (Chibnik, 2010).  However, this is done in a sample of patients with LN, therefore in general SLE population, the performance may not be as well, and we imposed additional prescription evidence. |
| Pelletier, 2009 | N=15,590 SLE patients identified from the IMS claims database, 2007 | Aged ≥18 years,  ≥2 claims for SLE (ICD-9 710.0) during a 6-month pre-index period through 3 months post-index), and continuous enrollment 6 months before and 12 months after the index date, and  ≥1 prescription fill for an immunosuppressive /disease-modifying antirheumatic drug (DMARD), antimalarial agent, NSAID/cyclooxygenase (COX)-2 inhibitor, or other SLE-related treatment. | We included commonly used medications for SLE as in most other studies above, but did not include the list of NSAIDs and other SLE-related treatment (eg, opioid and combination  analgesic, antianxiety, antihyperlipidemic, antihypertensive agent, bisphosphonate, vitamin D) etc by (Palletier, 2009) given the widespread use of these (including non-prescription) and the non-specific nature of their indications relative to SLE, as well as more stringent criteria of >=3 outpatient claims. |
| Jorge, 2019 | N=1322 patients with at least 1 SLE diagnosis code from the primary data source: Partners HealthCare Biobank, a large cohort of consented subjects with a biospecimen repository linked with a centralized longitudinal EHR database, the Research Patient Data Repository (RPDR); the larger Partners HealthCare EHR population, which includes the Biobank population, as a validation cohort. | Of 400 randomly selected charts, we identified gold standard cases, classified as definite SLE, probable SLE, or non-SLE by detailed medical record review. Two rheumatologists (AJ and CF) independently reviewed the same initial 100 subjects, adjudicated discrepancies, and applied the same rules to the subsequent 300 subjects. Definite cases were defined as meeting 1997 ACR or 2012 SLICC Classification Criteria for SLE, and were diagnosed as SLE by their rheumatologist1997 ACR or 2012 SLICC Classification Criteria | The present study does not have access to patients’ charts in the HIPAA compliant, de-identified databases. Therefore, the EHR based algorithm is not applicable to the present study.  As noted in (Jorge, 2019), fulfillment of ACR or SLICC criteria was not a requirement to define a SLE case due to missing criteria data in many charts, even in SLE cases who were followed with a rheumatologist, thus reflecting regional differences in SLE classification and documentation.  Ruling out SLE cases due to lack of available data for access would result in reduced sensitivity, which may not be a serious concern for an association analysis, but is not desirable for the prevalence estimation in the present study. |

# Additional Figures

## Additional Figure SF1. Age-, sex-specific SLE prevalence, 2016


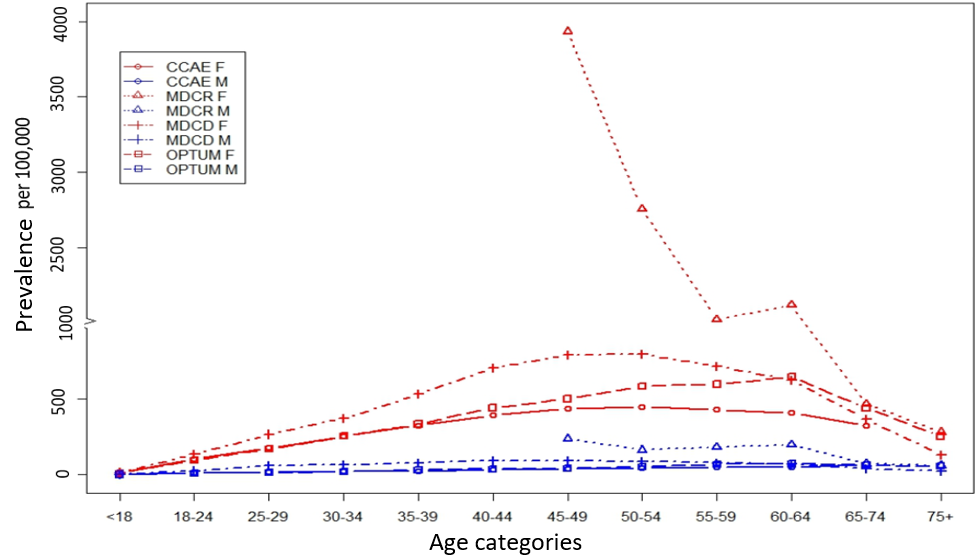


## Additional Figure SF2. Female: Male ratio of SLE prevalence by age, 2016

# Additional References

Aringer M, Costenbader K, Daikh D, et al. 2019a. European League against Rheumatism/ American College of rheumatology classification criteria for systemic lupus erythematosus. Ann Rheum Dis 2019;78:1151–9.

Aringer M, Costenbader KH, Dorner T, Johnson SR. 2019b. Difference between SLE classification and diagnosis and importance of attribution. Response to: 'Do the 2019 EULAR/ACR SLE classification criteria close the door on certain groups of SLE patients?' by Chi et al. Ann Rheum Dis. 2019.

Chibnik LB, Massarotti EM, Costenbader KH. Identification and validation of lupus nephritis cases using administrative data. *Lupus*. 2010;19(6):741-743.

Chung H, Deshpande G, Zolotarjova J, Quimbo RA, Kern DM, Cochetti PT, Willey VJ. Health Plan Enrollment and Disenrollment of Individuals With and Without Established Chronic Disease in a U.S. Commercially Insured and Medicare Advantage Population. J Manag Care Spec Pharm. 2019 May;25(5):612-620. doi: 10.18553/jmcp.2019.25.5.612. PMID: 31039058.

Dall'Era M, Cisternas MG, Snipes K, Herrinton LJ, Gordon C, Helmick CG. The incidence and prevalence of systemic lupus erythematosus in San Francisco County, California: the California lupus surveillance project. Arthritis & rheumatology. 2017 Oct;69(10):1996‐2005.

Feldman CH, Hiraki LT, Liu J, et al. Epidemiology and sociodemographics of systemic lupus erythematosus and lupus nephritis among US adults with Medicaid coverage, 2000-2004. *Arthritis Rheum*. 2013;65(3):753-763.

Ferucci ED, Johnston JM, Gaddy JR, Sumner L, Posever JO, Choromanski TL, et al. Prevalence and incidence of systemic lupus erythematosus in a population-based registry of American Indian and Alaska Native people, 2007-2009. Arthritis Rheumatol. 2014;66(9):2494-502.

Gergianaki I, Bertsias G. Systemic Lupus Erythematosus in Primary Care: An Update and Practical Messages for the General Practitioner. Frontiers in medicine. 2018;5:161.

Hochberg MC. Updating the American College of Rheumatology revised criteria for the classification of systemic lupus erythematosus. Arthritis Rheum 1997;40:1725

Izmirly PM, Wan I, Sahl S, Buyon JP, Belmont HM, Salmon JE, et al. The Incidence and Prevalence of Systemic Lupus Erythematosus in New York County (Manhattan), New York: The Manhattan Lupus Surveillance Program. Arthritis Rheumatol. 2017;69(10):2006-17.

Jorge A, Castro VM, Barnado A, et al. Identifying lupus patients in electronic health records: Development and validation of machine learning algorithms and application of rule-based algorithms. Semin Arthritis Rheum. 2019;49(1):84-90.

Kan H, Nagar S, Patel J, Wallace DJ, Molta C, Chang DJ. Longitudinal Treatment Patterns and Associated Outcomes in Patients With Newly Diagnosed Systemic Lupus Erythematosus. Clin Ther. 2016;38(3):610-24.

Klein RJ, Schoenborn CA. Age adjustment using the 2000 projected U.S. population. January 2001. URL: <https://www.cdc.gov/nchs/data/statnt/statnt20.pdf>.

Lam NC, Ghetu MV, Bieniek ML. Systemic Lupus Erythematosus: Primary Care Approach to Diagnosis and Management. Am Fam Physician. 2016;94(4):284-294.

Lim SS, Bayakly AR, Helmick CG, Gordon C, Easley KA, Drenkard C. The incidence and prevalence of systemic lupus erythematosus, 2002–2004: The Georgia Lupus Registry. Arthritis & rheumatology. 2014 Feb;66(2):357‐68.

Petri M, Orbai AM, Alarco´ n GS et al. Derivation and validation of the Systemic Lupus International Collaborating Clinics classification criteria for systemic lupus erythematosus. Arthritis Rheum 2012;64:267786.

Somers EC, Marder W, Cagnoli P, Lewis EE, DeGuire P, Gordon C, Helmick CG, Wang L, Wing JJ, Dhar JP, Leisen J. Population‐based incidence and prevalence of systemic lupus erythematosus: the Michigan Lupus Epidemiology and Surveillance program. Arthritis & rheumatology. 2014 Feb;66(2):369‐78.

Steiman AJ, Urowitz MB, Ibañez D, Papneja A, Gladman DD. Prolonged clinical remission in patients with systemic lupus erythematosus. *J Rheumatol*. 2014;41(9):1808-1816.

Tan EM, Cohen AS, Fries JF, Masi AT, McShane DJ, Rothfield NF, et al. The 1982 revised criteria for the classification of systemic lupus erythematosus. Arthritis and rheumatism. 1982;25(11):1271-7.

Tenny S, Hoffman MR. Prevalence. 2020 Jul 10. In: StatPearls [Internet]. Treasure Island (FL): StatPearls Publishing; 2020 Jan–. PMID: 28613617.

Thong B, Olsen NJ. Systemic lupus erythematosus diagnosis and management. Rheumatology. 2017;56(suppl_1): i3-i13.

Voss EA, Makadia R, Matcho A, et al. Feasibility and utility of applications of the common data model to multiple, disparate observational health databases. J Am Med Inform Assoc. 2015; 22: 553-64.
